# Management and clinical outcome of myocardial infarction in Kosovo: A cross‐sectional study

**DOI:** 10.1002/hsr2.70122

**Published:** 2024-10-16

**Authors:** Arlind Batalli, Michael Henein, Afrim Poniku, Pranvera Ibrahimi, Edita Pllana‐Pruthi, Shpend Elezi, Faik Shatri, Genc Abdyli, Artan Bajraktari, Rona Karahoda, Hamza Selmani, Ibadete Bytyçi, Gani Bajraktari

**Affiliations:** ^1^ Clinic of Cardiology University Clinical Centre of Kosova Prishtina Kosovo; ^2^ Medical Faculty University of Prishtina Prishtina Kosovo; ^3^ Department of Public Health and Clinical Medicine Umeå University Sweden; ^4^ Research Unit, Heimerer College, 10000 Prishtina Kosovo

**Keywords:** acute myocardial infarction, Kosovo, mortality, primary PCI

## Abstract

**Background and Aims:**

Myocardial infarction (MI) is *a major* cause of mortality worldwide, irrespective of its presentation as non‐ST‐segment elevation MI (NSTEMI) or ST‐segment elevation MI (STEMI). The objective of this study was to assess national results of management and clinical outcome of acute MI patients in Kosovo.

**Methods:**

This cross‐sectional descriptive study, conducted at the Clinic of Cardiology of the University Clinical Center of Kosovo, in Prishtina, included all patients hospitalized with acute MI over a period of 7 years (2014‐2020). The primary outcome of the study was in‐hospital mortality.

**Results:**

Among 7353 admitted patients with acute MI (mean age 63 ± 12 years, 29% female) and according to the final diagnosis, 4436 (59.4%) patients had STEMI, and 2987 (40.6%) NSTEMI. More patients with STEMI received primary percutaneous intervention (PPCI) than those with NSTEMI (50% vs. 41%, *p* < 0.001). In‐hospital mortality was higher in no PPCI patients compared to PPCI both in NSTEMI (10.7% vs. 2.6%, *p* < 0.001) and STEMI (20.9% vs. 6.8%, *p* < 0.001). Age ≥65 years [0.399 (0.267–0.597), *p* ˂ 0.001], hemoglobin level [0.889 (0.815–0.970), *p* = 0.008], STEMI [0.491 (0.343–0.704), *p* ˂ 0.001], lack of PPCI [2.636 (1.798–3.866), p ˂ 0.001], cardiogenic shock [0.002 (0.001–0.006), *p* < 0.001], reduced left ventricular ejection fraction (LV EF) [0.966 (0.951–0.980), *p* < 0.001], and heart rate at admission [1.009 (1.000–1.017), *p* = 0.047], independently predicted mortality. In STEMI, cardiogenic shock (*p* ˂ 0.001), lack of PPCI (*p* = 0.006), female gender (*p* = 0.01), and low LV EF (*p* = 0.04) predicted mortality but age ≥65 years (*p* = 0.02), female gender (*p* = 0.02), low LV EF (*p* = 0.007), and low hemoglobin (*p* = 0.04) predicted mortality in NSTEMI.

**Conclusion:**

Between 2014 and 2020, half of patients with acute MI were not treated with PPCI, who had high mortality, particularly when presenting with STEMI. Age, cardiogenic shock, anemia, low LV EF, STEMI and no PPCI independently predicted mortality. Cardiogenic shock and lack of PPCI independently predicted mortality, only in STEMI.

## Key learning points

*What is already known:*

- Acute MI is a worldwide health problem with a high mortality.
- Numerous national registries, worldwide, do exist and many include patients with acute MI, which results influenced to health care in these patients in different countries.
- Kosovo, as the youngest European country has not national data on the incidence and related mortality of acute MI.

*What this study adds:*

- This study represents the largest real population survey of almost all patients referred with acute MI from all regional hospitals of Kosovo, to our heart center, for optimum treatment.
- Between 2014 and 2020, half of patients with acute MI were not treated with PPCI, who had high mortality, particularly when presenting with STEMI.
- Age, cardiogenic shock, anemia, low LV EF, STEMI and no PPCI independently predicted mortality. Cardiogenic shock and lack of PPCI independently predicted mortality, only in STEMI.

## 1 INTRODUCTION

Acute myocardial infarction (MI) is a worldwide health problem with a high mortality.^1^ Despite the significant investment in its diagnosis and in early treatment including invasive procedures, acute MI related mortality remains high, even in developed countries.^2^ Many factors influence acute MI related mortality including cardiovascular risk factors, clinical presentation and the type of treatment patients receive.^3^, ^4^, ^5^ Another important factor is the great difficulties with low‐income and limited free health services in developing countries which compromise routine procedures^6^, ^7^ and evidence‐based treatments of acute MI.^8^, ^9^, ^10^, ^11^ This results in significant difference in acute MI related mortality between countries.^12^, ^13^, ^14^ Studies have also shown differences in risk related clinical outcome of these patients, with the most important predictors of mortality being older age, female gender, renal failure, high blood pressure, severe heart failure, previous CABG, and reduced left ventricular ejection fraction.^15^, ^16^

Identifying predictors of in‐hospital mortality is important for health professionals and health policy makers since they play a pivotal role in improving patients' diagnosis and treatment.^17^

Kosovo is one of the countries which face several difficulties in organizing structured strategy for optimum management of acute MI including diagnosis and PPCI in public hospitals. The University Clinical Centre of Kosovo is the only public hospital in Kosovo that performed primary PCI in patients with acute MI during the study period. In this interventional center all patients with acute MI referred from Prishtina district health institutions were treated, and also those referred from six regional public hospitals. This center started primary PCI in 2014, but only during daily working hours, and from 2018 it started providing the primary PCI service to the whole country 24 h/day. This management shortage is also contributed to by the lack of national data on the incidence and related mortality of acute MI in Kosovo which has never been optimally searched and documented. The aim of this study therefore, was to fill the gap of these knowledge by assessing the clinical outcome of AMI patients and identifying predictors of in‐hospital mortality in Kosovo.

## 2 METHODS

This study was conducted at the Clinic of Cardiology of the University Clinical Center of Kosovo, in Prishtina, using a cross‐sectional descriptive analysis. All patients hospitalized with acute MI (a total of 7353 patients) over a period of 7 years (from 1stJanuary 2014 to 31st December 2020) are included in the study. Data was collected using patients’ records stored in the hospital archive, and the results of the invasive diagnostic and interventional results were also registered, in all patients with acute MI who were diagnosed based on conventional electrocardiogram criteria and raised myocardial biomarkers. The data collection was performed after receiving approval from the Ethics Council of the Kosovo Doctors Chamber. Patients were divided into two groups based on the ST‐segment elevation at admission: 1) with non‐ST‐segment elevation myocardial infarction (NSTEMI) and 2) with ST‐segment elevation myocardial infarction (STEMI).^9^ In‐hospital mortality was defined as death from the time of patient's admission until discharge.

### 2.1 Statistical analysis

Patients' clinical characteristics and the events of in‐hospital mortality were collected retrospectively from the patients' medical data stored in the hospital archive, which then underwent thorough checking followed by many statistical analyses. Continuous variables are presented as mean (SD) and categorical variables as frequencies and percentages. Logistic regression analysis was performed to determine the predictors of in‐hospital mortality among patients with acute MI, as well as in two groups, STEMI and NSTEMI. We established a priori level of significance at α = 0.05. All statistical tests were two‐sided. This criterion ensured that results were considered statistically significant if *p* < 0.05, indicating a low probability (less than 5%) that observed effects were due to random chance. Statistical analyses were performed using IBM SPSS Statistics for Windows Operating System, version 24.0 software (IBM Corp.).

## 3 RESULTS

In total, 7353 patients were admitted with acute MI (mean age 63 ± 12 years, 29% female) during the seven years period (2014−2022). According to the final discharge diagnosis, 4366 (59.4%) patients were identified as having STEMI and 2987 (40.6%) patients as having NSTEMI. Of the group of patients as a whole, 1188 (16%) were transferred to another primary PCI hospital, and the 84% were managed at our Center.

### 3.1 STEMI versus NSTEMI patients

**Figure 1:**
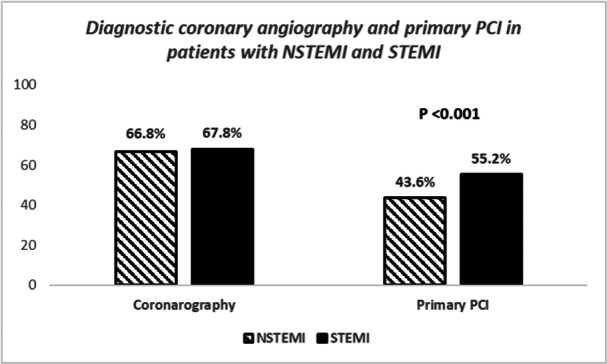
The coronary angiography and primary PCI rate in patients with STEMI and NSTEMI.

Patients’ age was not different between the two groups of patients (63 ± 11 vs. 64 ± 12 years, *p* = 0.08), but the NSTEMI cohort had less smokers (48.3% vs. 54%, *p* < 0.001), more diabetics (37.8% vs. 33.6%, *p* < 0.001), more hypertensives (69.6% vs. 63%, *p* < 0.001), more patients with family history for CAD (40% vs 38%, *p* = 0.009), and more females (32% vs 27%, *p* < 0.001) compared to the STEMI cohort (Table 1). At admission, patients with NSTEMI had lower glucose (9.2 ± 5 vs. 9.8 ± 6 mmol/L, *p* < 0.001), and higher triglycerides (2.0 ± 1.2 vs. 1.88 ± 1.3 mmol/L, *p* = 0.001) levels compared with STEMI patients, whereas cholesterol (4.8 ± 1.6 vs. 4.5 ± 1.5 mmol/L, *p* = 0.94), urea (9.2 ± 6 vs. 9.1 ± 6 mg/dL, *p* = 0.652), creatinine (118 ± 74 vs. 117 ± 73 μmol/L, *p* = 0.63), and hemoglobin (13.7 ± 3.4 vs. 13.7 ± 3.0 g/dL, *p* = 0.94) were not different between groups (Table 1). Also at admission, patients with NSTEMI had less atrial fibrillation (3.9% vs. 5.0%, *p* = 0.04), less left bundle branch block (2.6% vs. 5.3%, *p* < 0.001), higher left ventricular ejection fraction (51.6 ± 9% vs. 49.8 ± 9%, *p* < 0.001) and less cardiogenic shock (2.1% vs. 4.9%, *p* < 0.001) compared to STEMI patients (Table 1). There was no difference between the two cohorts with respect to the presence of significant CAD on diagnostic coronary angiography (66.8% vs. 67.8%, *p* = 0.40), but patients with NSTEMI underwent less PPCI compared with STEMI patients (43.6% vs. 55.2%, *p* < 0.001) (Table 2, Figure 1).

**Table 1:** Clinical and biochemical data in patients with acute myocardial infarction.

	All included patients	Patients with NSTEMI	Patients with STEMI	
Variable	**(*n*** = 7353**)**	**(*n*** = 2987**)**	**(*n*** = 4366**)**	*P* value
Age (years)	63 ± 12	63 ± 11	64 ± 12	0.08
Female (%)	28.7	31.7	26.6	˂0.001
Smoking (%)	51.7	48.3	54	˂0.001
Diabetes (%)	35.3	37.8	33.6	˂0.001
Hypercholesterolemia (%)	40.1	39.4	40.1	0.099
Arterial hypertension (%)	65.6	69.6	63	<0.001
Family history for CAD (%)	38.1	39.9	36.9	0.009
Atrial fibrillation (%)	4.4	3.9	5.0	0.04
Left bundle branch block (%)	3.7	2.6	5.3	<0.001
Cardiogenic shock (%)	3.7	2.1	4.9	<0.001
Fasting glucose (mmol/L)	9.5 ± 5.5	9.2 ± 5	9.8 ± 6	<0.001
Total cholesterol (mmol/L)	4.8 ± 1.6	4.8 ± 1.6	4.5 ± 1.5	0.94
Triglycerides (mmol/L)	1.9 ± 1.3	2.0 ± 1.3	1.88 ± 1.2	0.001
Creatinine (μmol/L)	118 ± 74	118 ± 74	117 ± 73	0.63
Urea (mg/dL)	9.2 ± 6	9.2 ± 6	9.1 ± 6	0.65
Hemoglobin (g/dl)	13.7 ± 3.2	13.7 ± 3.4	13.7 ± 3	0.94
Heart rate at admission (beats/min)	82.6 ± 23	82.5 ± 26	82.6 ± 20	0.82
Left ventricular ejection fraction (%)	50.6 ± 9	51.6 ± 9	49.8 ± 9	<0.001

Abbreviations: CAD, coronary artery disease; NSTEMI, Non‐ST‐segment elevation myocardial infarction; STEMI, ST‐segment‐elevation myocardial infarction.

**Table 2:** Invasive treatment of patients with acute myocardial infarction.

	All included patients	Patients with NSTEMI	Patients with STEMI	
Variable	**(*n*** = 7353**)**	**(*n*** = 2987**)**	**(*n*** = 4366**)**	*P* value
Coronary angiography (%)	67.3	66.8	67.8	0.40
Primary Percutaneous Intervention (%)	50.1	43.6	55.2	<0.001
In‐hospital mortality (%)	10.5	7.2	13.1	<0.001

Abbreviations: NSTEMI, Non‐ST‐segment elevation myocardial infarction; STEMI, ST‐segment‐elevation myocardial infarction.

### 3.2 In‐hospital mortality

**Figure 2:**
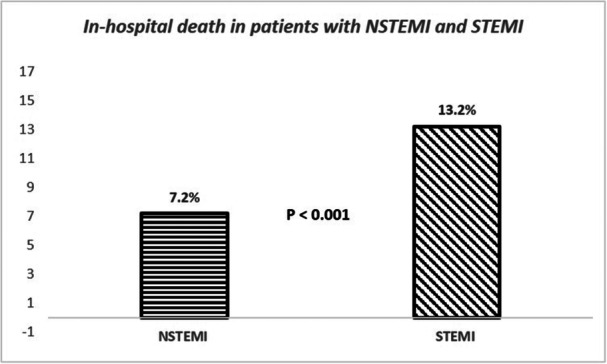
The coronary angiography and primary PCI rate in pateients with STEMI and NSTEMI.

In‐hospital mortality in the patient group who were not transferred to the 24 h PPCI center was 10.6%, which was significantly higher in STEMI compared to NSTEMI patients (13.2% vs. 7.2%, *p* < 0.001) (Figure 2). Also, mortality was higher in patients who did not undergo reperfusion by PPCI, both in NSTEMI (10.7% vs. 2.6%, *p* < 0.001) and STEMI (20.9% vs. 6.8%, *p* < 0.001), compared to those who underwent PPCI.

**Figure 3:**
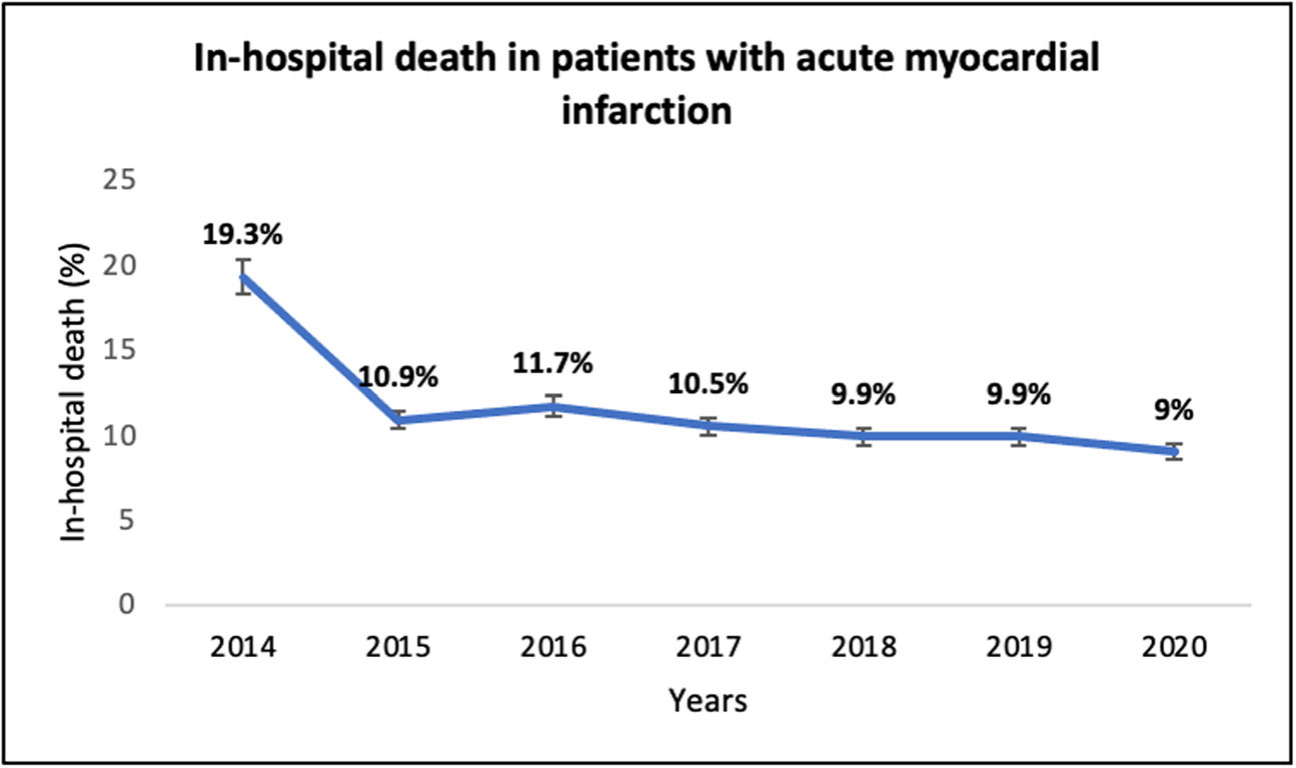
Number of hospitalized patients with acute myocardial infarction and in‐hospital mortality rate during the seven years of the study.

**Figure 4:**
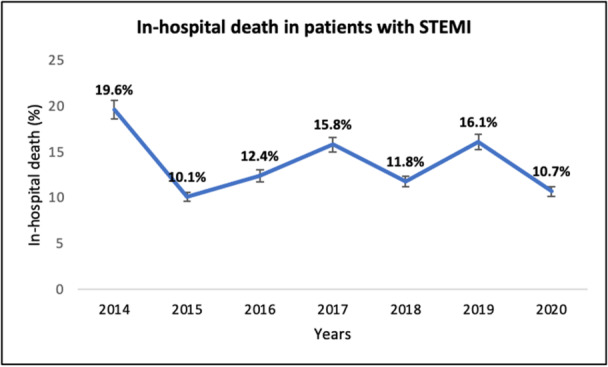
Number of hospitalized patients with STEMI and in‐hospital mortality rate during the seven years of the study.

In‐hospital mortality was very high (19.3%) before introducing primary PCI in 2015, dropping significantly (*p* < 0.001) over the years, but not noticeably after commencing 24 h a day/7 day a week primary PCI in 2018 (*p* = 0.26) (Figure 3). The same scenario happened in STEMI patients (Figure 4).

### 3.3 Predictors of in‐hospital mortality in patients with acute MI (Table 3)

In univariate analysis, age ≥65 years old, female gender, diabetes, smoking, family history for CAD, cardiogenic shock, heart rate at admission, atrial fibrillation, LV EF, low hemoglobin, hyperglycemia at admission, raised urea and/or creatinine, STEMI, lack of coronary angiography and PPCI (*p* < 0.001 for all), as well as raised cholesterol level (*p* = 0.021) were predictors of in‐hospital mortality. The multivariate analysis identified age ≥65 years [0.399 (0.267–0.597), *p* ˂ 0.001], low hemoglobin [0.889 (0.815–0.970), *p* = 0.008], STEMI [0.491 (0.343–0.704), *p* ˂ 0.001], lack of PPCI [2.636 (1.798–3.866), *p* ˂ 0.001], cardiogenic shock [0.002 (0.001–0.006), *p* < 0.001], low LV EF [0.966 (0.951–0.980), *p* < 0.001], and fast heart rate at admission [1.009 (1.000‐1.017), *p* = 0.047] as independent predictors of in‐hospital mortality.

**Table 3:** Predictors of in‐hospital mortality in patients with acute myocardial infarction.

	*Univariate predictors*			*Multivariate predictors*		
Variable	OR	(CI 95%)	*P* value	OR	(CI 95%)	*P* value
Age ≥65 years old	0.249	(0.207–0.300)	<0.001	0.399	(0.267–0.597)	<0.001
Gender	0.484	(0.410–0.571)	<0.001			
Arterial hypertension	1.061	(0.896–1.255)	0.493			
Diabetes mellitus	0.557	(0.474–0.655)	<0.001			
Smoking	1.805	(1.530–2.129)	<0.001			
Family anamnesis for CAD	1.370	(1.153–1.628)	<0.001			
Heart rate at admission	1.017	(1.013–1.021)	<0.001	1.009	(1.000–1.017)	0.047
Atrial fibrillation	0.315	(0.239–0.416)	<0.001			
LV ejection fraction	0.948	(0.940–0.957)	<0.001	0.966	(0.951–0.980)	<0.001
Hemoglobin	0.804	(0.766–0.844)	<0.001	0.889	(0.815–0.970)	0.008
Glycemia at admission	1.116	(1.101–1.132)	<0.001			
Cholesterol	0.876	(0.783–0.980)	0.021			
Triglycerides	0.959	(0.835–1.100)	0.546			
Urea	1.080	(1.067–1.093)	<0.001			
Creatinine	1.005	(1.004–1.006)	<0.001			
Presence of STEMI	0.510	(0.427–0.608)	<0.001	0.491	(0.343–0.704)	<0.001
Performed coronarography	4.206	(3.549–4.985)	<0.001			
Performed primary PCI	3.421	(2.840–4.120)	<0.001	2.636	(1.798–3.866)	<0.001
Cardiogenic shock	0.005	(0.003–0.007)	<0.001	0.002	(0.001–0.006)	<0.001

Abbreviations: CAD, coronary artery disease; PCI, percutaneous coronary intervention; STEMI, ST‐segment elevation myocardial infarction.

### 3.4 Predictors of mortality in STEMI and NSTEMI patients (Tables 4 & 5)

In patients with STEMI, cardiogenic shock (p˂0.00), lack of PPCI (*p* = 0.006), female gender (*p* = 0.01), and low LV EF (*p* = 0.04) independently predicted in‐hospital mortality. Respective predictors of mortality in NSTEMI were age ≥65 years (*p* = 0.02), female gender (*p* = 0.02), low LV EF (*p* = 0.007), and low hemoglobin (*p* = 0.04).

**Table 4:** Predictors of in‐hospital mortality in patients with NSTEMI.

	*Univariate predictors*			*Multivariate predictors*		
Variable	OR	(CI 95%)	*P* value	OR	(CI 95%)	*P* value
Age ≥65 years old	0.279	(0.199–0.392)	<0.001	0.269	(0.092–0.786)	0.02
Gender	0.559	(0.416–0.753)	<0.001	0.152	(0.032–0.715)	0.02
Arterial hypertension	1.061	(0.896–1.255)	0.49			
Diabetes mellitus	0.555	(0.413–0.745)	<0.001			
Smoking	1.579	(1.167–2.137)	0.003			
Family anamnesis for CAD	1.376	(1.008–1.879)	0.045			
Heart rate at admission	1.017	(1.013–1.021)	<0.001			
Atrial fibrillation	0.275	(0.175–0.432)	<0.001			
LV ejection fraction	0.941	(0.924–0.958)	<0.001	0.953	(0.919–0.987)	0.007
Hemoglobin	0.755	(0.696–0.819)	<0.001	0.773	(0.608–0.983)	0.04
Glycemia at admission	1.125	(1.097–1.154)	<0.001			
Cholesterol	0.952	(0.786–1.152)	0.61			
Triglycerides	0.904	(0.697–1.174)	0.45			
Urea	1.080	(1.049–1.091)	<0.001			
Creatinine	1.003	(1.002–1.005)	<0.001			
Performed coronarography	5.140	(3.739–7.006)	<0.001			
Performed primary PCI	4.561	(3.065–6.786)	<0.001			
Cardiogenic shock	0.004	(0.002–0.012)	<0.001			

Abbreviations: CAD, coronary artery disease; STEMI, ST‐segment elevation myocardial infarction; PCI, percutaneous coronary intervention.

**Table 5:** Predictors of in‐hospital mortality in patients with STEMI.

	*Univariate predictors*			*Multivariate predictors*		
Variable	OR	(CI 95%)	*P* value	OR	(CI 95%)	*P* value
Age ≥65 years old	0.247	(0.197–0.309)	<0.001			
Gender	0.420	(0.343–0.515)	<0.001	0.457	(0.246–0.852)	0.01
Arterial hypertension	1.027	(0.836–1.262)	0.80			
Diabetes mellitus	0.588	(0.482–0.718)	<0.001			
Smoking	1.936	(1.583–2.368)	<0.001			
Family anamnesis for CAD	1.437	(1.162–1.777)	0.001			
Heart rate at admission	1.017	(1.013–1.021)	<0.001			
Atrial fibrillation	0.336	(0.232–0.487)	<0.001			
LV ejection fraction	0.955	(0.945–0.964)	<0.001	0.975	(0.952–0.999)	0.04
Hemoglobin	0.828	(0.779–0.881)	<0.001			
Glycemia at admission	1.126	(1.106–1.146)	<0.001			
Cholesterol	0.833	(0.725–0.957)	0.010			
Triglycerides	1.006	(0.855–1.182)	0.95			
Urea	1.102	(1.083–1.121)	<0.001			
Creatinine	1.008	(1.006–1.009)	<0.001			
Performed coronarography	4.004	(3.265–4.911)	<0.001			
Performed primary PCI	3.586	(2.891–4.449)	<0.001	2.243	(1.256–4.004)	0.006
Cardiogenic shock	0.006	(0.003–0.011)	<0.001	0.006	(0.002–0.018)	<0.001

Abbreviations: CAD, coronary artery disease; PCI, percutaneous coronary intervention; STEMI, ST‐segment elevation myocardial infarction.

## 4 DISCUSSION

The aim of this observational study was to investigate the management policy and in‐hospital outcome of patients presenting with acute MI, admitted to the University Clinical Centre of Kosova, the only tertiary healthcare center in Kosovo, over the course of seven years. This study represents the largest real population survey of almost all patients referred with acute MI from all regional hospitals of Kosovo, to our heart center, for optimum treatment, hence patients recruitment deserves to be described as consecutive.

### 4.1 Findings

Our data analysis shows that in Kosovo, females suffered fewer MIs than males. The majority of cases presenting with acute MI had STEMI who less commonly had diabetes, arterial hypertension and family history for CAD, but smoked more, compared to those presenting with NSTEMI. In addition, females were more prevalent among the NSTEMI compared to STEMI patients. Patients with NSTEMI underwent less PPCI compared with STEMI patients.

In‐hospital mortality in patients with acute MI who were not transferred to the 24‐hour PPCI center was 10.6%, which was significantly higher in STEMI compared to NSTEMI patients, and this was particularly seen in patients who did not undergo reperfusion by PPCI, both in STEMI and NSTEMI patients. Age ≥65 years, low hemoglobin level, diagnosis of STEMI, lack of PPCI, cardiogenic shock, low LV EF, and slow heart rate at admission independently predicted in‐hospital mortality in the group of patients with acute MI, as a whole. While cardiogenic shock, lack of PPCI, female gender and low LV EF independently predicted in‐hospital mortality in STEMI patients. In NSTEMI patients age ≥65 years, female gender, low LV EF and reduced level of hemoglobin were the independent predictors.

### 4.2 Data interpretation

Our analysis shows that in Kosovo, almost half of the patients presenting with acute MI did not undergo primary PCI during the 7 years of the study period. This data shows, probably, the worst percentage of optimally treated patients with primary PCI, based on the available European clinical guidelines at the time. Several factors could have contributed to the low treatment adherence to the evidence‐based facts. The historical past of Kosovo as the youngest country in Europe, lack of health insurance, late establishment of interventional cardiology and shortage of staff expertise, national financial constraints, and lack of a proper strategic plan from the health care institutions. These are just some of the very obvious reasons behind the significant difference between Kosovo acute MI treatment in comparison with the then‐published European recommendations. Of course, the extent of the contribution of individual factors cannot be ignored and equally cannot be numerically estimated.

The most important clinical outcome of the above acute MI treatment strategy in Kosovo is in‐hospital mortality which is expected to be influenced by the same factors mentioned above which significantly impacted patients with STEMI much more than those with NSTEMI. It should be mentioned that the mortality rate in discussion is, at least, three times over and above its respective mortality in the European Society of Cardiology member countries (13.2% vs. 4.4%)^14^, ^18^ in patients with STEMI. In fact, similar high mortality rate of patients with acute MI has been shown in several neighboring countries at the time when primary PCI was not available, thus supporting the above explanation.

Following the management pattern seen in other countries, as primary PCI was first introduced in the only tertiary health care center in Kosovo in 2014, and 4 years later the service became a routine weekly 24 h/day in‐hospital mortality significantly dropped. Similar, high mortality of patients with acute MI has been shown in several neighboring countries when PPCI treatment was not available.^19^, ^20^ However, this positive response should not be seen as contradicting the remaining global pattern of high mortality rate, especially in older patients.^21^

### 4.3 Study limitations

We did not recruit all Kosovo patients with acute MI, but we included all those admitted to the University Hospital, the only public hospital in Kosovo where primary PCI was available, in whom the diagnosis of acute MI was confirmed. We could have missed some patients with acute MI in the territory of Kosovo that were not hospitalized and/or died at home, a factor that might impact the calculated percentage. We did not assess the impact of other diseases these patients might have had but focused on cardiovascular conventional risk factors and their potential effect on clinical outcome.

## 5 CONCLUSION

This acute MI audit from Kosovo reflects the natural history and clinical outcome of a large population of patients admitted to hospitals irrespective of the pattern of presentation. It also portraits the significant decline in in‐hospital mortality following the beneficial effect of nonsurgical revascularization and following conventional international guidelines.

## AUTHOR CONTRIBUTIONS

**Arlind Batalli**: Conceptualization; Methodology; Validation; Visualization; Writing—original draft. **Michael Henein**: Conceptualization; Supervision; Validation; Writing—review and editing. **Afrim Poniku**: Formal analysis; Investigation; Methodology; Project administration; Supervision; Validation; Visualization. **Pranvera Ibrahimi**: Conceptualization; Data curation; Formal analysis; Methodology; Supervision; Validation; Visualization. **Edita‐Pllana**: Data curation; Funding acquisition; Investigation; Methodology; Project administration; Resources. **Shpend Elezi**: Conceptualization; Validation; Writing—review and editing. **Faik Shatri**: Data curation; Investigation; Project administration; Resources; Validation; Visualization. **Genc Abdyli**: Data curation; Formal analysis; Funding acquisition; Investigation; Methodology; Resources; Software. **Artan Bajraktari**: Conceptualization; Data curation; Formal analysis; Investigation; Methodology; Writing—original draft. **Rona Karahoda**: Data curation; Formal analysis; Methodology; Software; Validation. **Hamza Selmani**: Data curation; Funding acquisition; Investigation; Methodology; Project administration; Resources. **Ibadete Bytyçi**: Methodology. **Gani Bajraktari**: Conceptualization; Data curation; Formal analysis; Investigation; Methodology; Project administration; Supervision; Validation; Visualization; Writing—original draft; Writing—review and editing.

## CONFLICT OF INTEREST STATEMENT

None declared.

## TRANSPARENCY STATEMENT

The lead author Afrim Poniku affirms that this manuscript is an honest, accurate, and transparent account of the study being reported; that no important aspects of the study have been omitted; and that any discrepancies from the study as planned (and, if relevant, registered) have been explained.

## Data Availability

The data underlying this article will be shared on reasonable request to the corresponding author. All authors have read and approved the final version of the manuscript [CORRESPONDING AUTHOR or MANUSCRIPT GUARANTOR] had full access to all data in this study and takes complete responsibility for the integrity of the data and the accuracy of the data analysis. There are no supporting source/financial relationships for this study.
